# Deletion of the Mitochondrial Superoxide Dismutase *sod-2* Extends Lifespan in *Caenorhabditis elegans*

**DOI:** 10.1371/journal.pgen.1000361

**Published:** 2009-02-06

**Authors:** Jeremy M. Van Raamsdonk, Siegfried Hekimi

**Affiliations:** Department of Biology, McGill University, Montreal, Quebec, Canada; Stanford University Medical Center, United States of America

## Abstract

The oxidative stress theory of aging postulates that aging results from the accumulation of molecular damage caused by reactive oxygen species (ROS) generated during normal metabolism. Superoxide dismutases (SODs) counteract this process by detoxifying superoxide. It has previously been shown that elimination of either cytoplasmic or mitochondrial SOD in yeast, flies, and mice results in decreased lifespan. In this experiment, we examine the effect of eliminating each of the five individual *sod* genes present in *Caenorhabditis elegans*. In contrast to what is observed in other model organisms, none of the *sod* deletion mutants shows decreased lifespan compared to wild-type worms, despite a clear increase in sensitivity to paraquat- and juglone-induced oxidative stress. In fact, even mutants lacking combinations of two or three *sod* genes survive at least as long as wild-type worms. Examination of gene expression in these mutants reveals mild compensatory up-regulation of other *sod* genes. Interestingly, we find that *sod-2* mutants are long-lived despite a significant increase in oxidatively damaged proteins. Testing the effect of *sod-2* deletion on known pathways of lifespan extension reveals a clear interaction with genes that affect mitochondrial function: *sod-2* deletion markedly increases lifespan in *clk-1* worms while clearly decreasing the lifespan of *isp-1* worms. Combined with the mitochondrial localization of SOD-2 and the fact that *sod-2* mutant worms exhibit phenotypes that are characteristic of long-lived mitochondrial mutants—including slow development, low brood size, and slow defecation—this suggests that deletion of *sod-2* extends lifespan through a similar mechanism. This conclusion is supported by our demonstration of decreased oxygen consumption in *sod-2* mutant worms. Overall, we show that increased oxidative stress caused by deletion of *sod* genes does not result in decreased lifespan in *C. elegans* and that deletion of *sod-2* extends worm lifespan by altering mitochondrial function.

## Author Summary

In this paper, we examine the oxidative stress theory of aging using *C. elegans* as a model system. This theory proposes that aging results from the accumulation of molecular damage caused by reactive oxygen species (ROS). To test this theory, we examined the effect of deleting each of the five individual superoxide dismutase (SOD) genes on lifespan and sensitivity to oxidative stress. Since SOD acts to detoxify ROS, the oxidative stress theory predicts that deletion of *sod* genes should increase oxidative stress and decrease lifespan. However, in contrast to yeast, flies, and mice, where loss of either cytoplasmic or mitochondrial SOD results in decreased lifespan, we find that none of the *sod* deletion mutants in *C. elegans* exhibits a shortened lifespan despite increased sensitivity to oxidative stress. Surprisingly, we find that *sod-2* mutant worms have extended lifespan and even worms with the primary cytoplasmic, mitochondrial, and extracellular *sod* genes deleted can live longer than wild-type worms. By examining genetic interactions with other genes known to extend lifespan and by comparing the phenotype of worms lacking *sod-2* to that of known long-lived mitochondrial mutants such as *clk-1* or *isp-1*, we provide evidence that the loss of *sod-2* extends lifespan through alteration of mitochondrial function.

## Introduction

The oxidative stress theory of aging proposes that reactive oxygen species (ROS) generated by normal metabolism cause damage to macromolecules within the cell and that the accumulation of this damage over time leads to cellular dysfunction and eventually organismal death [1]–[3]. The majority of ROS present in the cell is thought to be generated in the mitochondria. In order to counteract this process, cells have a number of defense mechanisms which serve to detoxify ROS. Superoxide dismutase (SOD) is a detoxification enzyme that converts superoxide to hydrogen peroxide, which can subsequently be converted to water [4].

The oxidative stress theory of aging predicts that loss of SOD activity should result in increased sensitivity to oxidative stress, since the organism would be less able to detoxify ROS. This should, in turn, result in a shortened lifespan. This is essentially what is observed in yeast, flies and mice for both cytoplasmic SOD (SOD1, CuZnSOD) and mitochondrial SOD (SOD2, MnSOD). In yeast, knocking out *sod1* has been shown to decrease clonal and replicative lifespan [5],[6] and accelerate chronological aging [7],[8]. In flies, knocking out *Sod1* decreases lifespan [9]. In mice, targeted inactivation of *Sod1* results in high oxidative stress and a 30% decrease in lifespan [10].

For SOD2, yeast knockouts show decreased chronological and replicative lifespan [6]–[8]. Reduction of *Sod2* in flies by either RNA interference (RNAi) or genetic deletion results in marked reductions in lifespan [11],[12]. In mice, *Sod2* knockouts exhibit high degrees of oxidative stress and neonatal or perinatal lethality [13],[14]. In contrast, loss of extracellular SOD (SOD3, EC-SOD) does not appear to impact lifespan despite an increased sensitivity to hyperoxia [15]. Thus, in support of the oxidative stress theory, the effect of deleting *Sod1* or *Sod2* in all three model species is increased oxidative stress and decreased lifespan or early lethality in the case of *Sod2* mice.

In contrast, lifespan in *C. elegans* may be relatively unaffected by decreased *sod* expression. Using an RNAi approach to knockdown either *sod-1* or *sod-2*, Yang *et al.* showed a mild decrease in lifespan with *sod-1* RNAi but no effect of *sod-2* RNAi, despite the fact that both knockdowns resulted in increased sensitivity to paraquat and an increase in oxidatively damaged proteins [16]. However, it is possible that if the RNAi did not completely abolish *sod* expression then the remaining low level of SOD activity is sufficient for normal lifespan.

Here, we examine the effect of eliminating SOD on lifespan and sensitivity to oxidative stress in *C. elegans* and thereby test the oxidative stress theory of aging. Whereas most organisms have only three SODs (one cytoplasmic, one mitochondrial and one extracellular), *C. elegans* has five *sod* genes [17]. *sod-1*, *sod-2* and *sod-4* encode the primary cytoplasmic, mitochondrial and extracellular SODs respectively [18]–[22] (equivalent to *Sod1*, *Sod2* and *Sod3* in mice). In addition, *sod-3* is expressed in the mitochondrial matrix and *sod-5* is expressed in the cytoplasm, thereby providing *C. elegans* with two cytoplasmic and two mitochondrial SODs [18],[23]. By examining *C. elegans* mutants with deletions in each of the five *sod* genes, we find that elimination of individual *sod* genes can increase sensitivity to oxidative stress but does not decrease lifespan. Furthermore, we find that *sod-2* mutant worms are long-lived and propose that their lifespan extension is due to an alteration of mitochondrial function.

## Results

### Elimination of Individual *sod* Genes Does Not Reduce Lifespan

**Figure 1:**
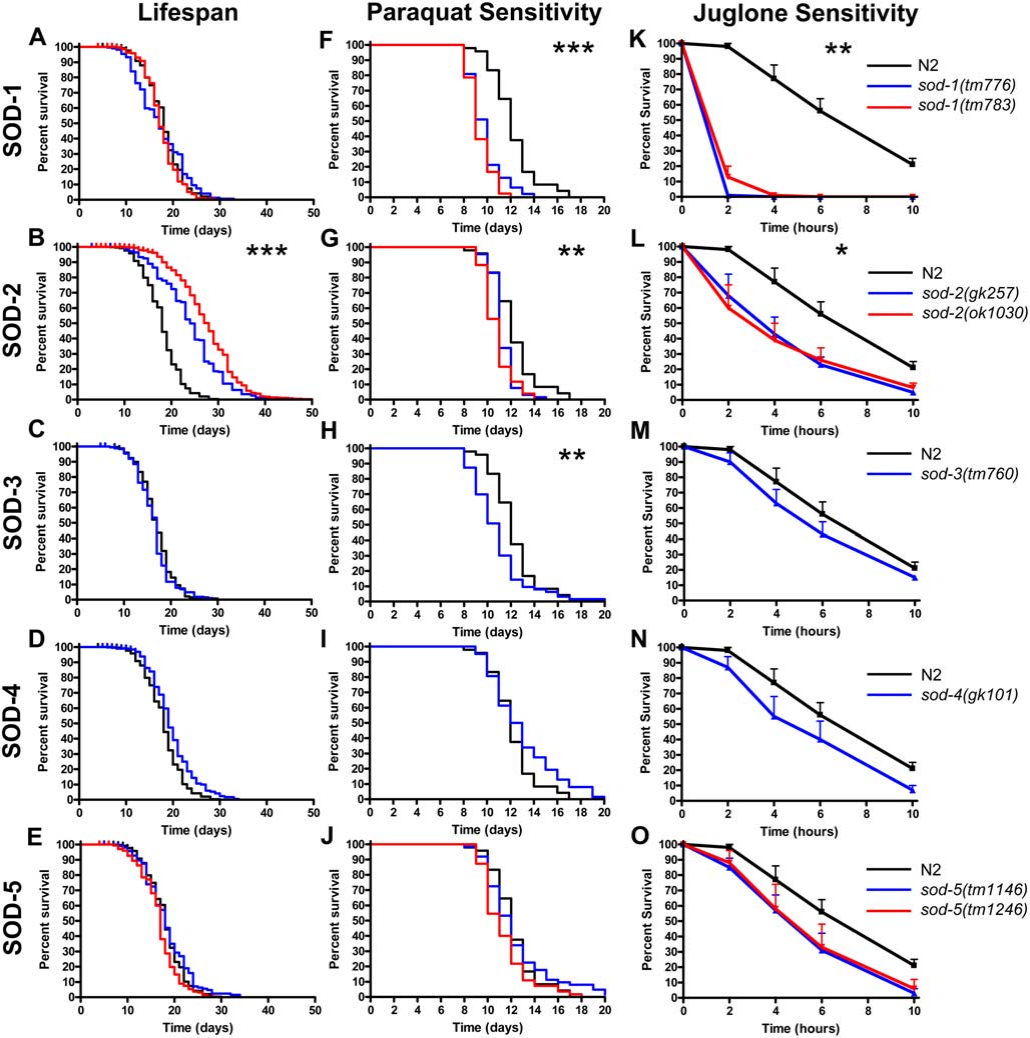
Deletion of Individual *sod* Genes Does Not Decrease Lifespan Despite Increasing Sensitivity to Oxidative Stress. A–E) *sod-1*(A), *sod-3*(C), *sod-4*(D) and *sod-5*(E) mutant worms live as long as wild-type N2 worms while *sod-2* mutant worms (B) live significantly longer than wild-type worms. F–J) *sod-1* (F), *sod-2* (G) and *sod-3* (H) mutant worms showed decreased survival on 4 mM paraquat plates compared to N2 worms, while *sod-4* (I) and *sod-5* (J) mutant worms survived as long as N2 worms. K–O) Similarly, *sod-1* (K) and *sod-2* (L) mutant worms showed decreased survival on 240 µM juglone plates compared to N2 worms, while the survival of *sod-3* (M), *sod-4* (N) and *sod-5* (O) mutant worms was not significantly different than N2 worms. Overall, *sod* deletion increases sensitivity to oxidative stress but does not decrease lifespan. * p<0.05, ** p<0.01, ***p<0.001.

The oxidative stress theory of aging predicts that increasing oxidative stress should result in decreased lifespan. To test this hypothesis, we assessed the lifespan of worms lacking each of the five individual *sod* genes in *C. elegans* (the location and size of the mutation for each allele tested are shown in Figure S1). For *sod-1*, *sod-2* and *sod-5* we assessed two independent alleles. We found that lifespan was not affected by the disruption of *sod-1*, *sod-3*, *sod-4* or *sod-5* (Figure 1A,C–E; all lifespan data is included in Table S1). This is particularly surprising in the case of *sod-1* since SOD-1 accounts for the majority of SOD activity in the cell [23]. In addition we found that deletion of *sod-2* resulted in a significant increase in lifespan (Figure 1B). This is also a surprising result given that SOD-2 is the primary SOD present in the mitochondrial matrix and the mitochondria is a major site of superoxide production in the cell.

To ensure that the lifespan extension in *sod-2* mutant worms resulted from the deletion of the *sod-2* gene we generated heteroallelic mutants. This was accomplished by crossing *sod-2(gk257)* males with *sod-2(ok1030)* hermaphrodites and following lifespan in the male offspring (since these must be cross progeny) and by crossing *sod-2(gk257)* males with either *dpy-17* (control) or *sod-2(ok1030);dpy-17* hermaphrodites and following the lifespan of the resulting non-dumpy hermaphrodite offspring. In both cases, we found that heteroallelic *sod-2(gk257)/sod-2(ok1030)* mutant worms lived significantly longer than their corresponding controls (Figure S2).

### Elimination of Individual *sod* Genes Results in Increased Sensitivity to Oxidative Stress

Since the loss of individual SODs failed to decrease lifespan, we next sought to determine whether the deletion of individual *sod* genes had an impact on oxidative stress. As it is currently not possible to accurately quantify the levels of ROS in worms, we used paraquat and juglone to assess sensitivity to oxidative stress as has been described in previous experiments [24]–[26]. Both of these compounds are reduced upon entry into the cell and are thought to induce oxidative stress by generating superoxide from oxygen during their subsequent reoxidation [27],[28].

To assess paraquat sensitivity, we examined the survival of 7 day old adult worms on plates containing 4 mM paraquat. We found that *sod-1* mutant worms were very sensitive to paraquat with all of the worms dying within one or two days (Figure 1F). We also found that *sod-2* and *sod-3* mutant worms were more sensitive to paraquat than wild-type worms although not as sensitive as *sod-1* mutant worms (Figure 1G,H). In contrast, *sod-4* and *sod-5* mutant worms showed similar survival to wild-type worms (Figure 1I,K)

In order to confirm our observation of increased sensitivity to oxidative stress, we assessed sensitivity to juglone. One day old worms were transferred to plates containing 240 µM juglone and survival was monitored for the following 10 hours. As with the paraquat assay, both *sod-1* deletion strains showed markedly increased sensitivity to oxidative stress as no worms survived to the 4 hour time point (Figure 1L). The *sod-2* deletion strains also showed increased sensitivity to juglone which was not as severe as the *sod-1* mutants (Figure 1M). In contrast, deletion of *sod-3*, *sod-4* or *sod-5* did not make worms significantly more sensitive to juglone-induced oxidative stress (Figure 1N–P).

Next, we assessed sensitivity to paraquat during development by exposing eggs to plates containing 0.2 mM paraquat and determining the latest developmental stage attained for each strain. While exposure to paraquat slowed development in all strains, including wild-type N2 worms, we found that all of the *sod* deletion mutants except for *sod-2* were able to develop to adulthood (Figure S3). The *sod-2* mutants were found to arrest at the L1 stage. Thus, *sod-2* mutant worms are the most sensitive of all of the *sod* deletion mutants to oxidative stress during development while *sod-1* mutant worms are the most sensitive in adulthood.

### Mild Compensatory Upregulation of *sod* mRNA in *sod* Deletion Mutants

**Figure 2:**
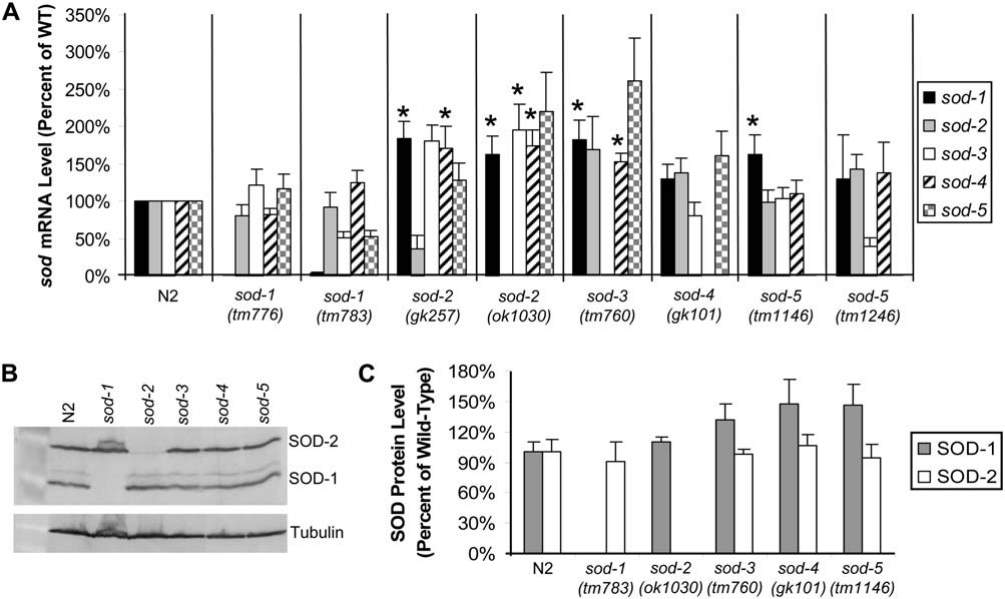
Deletion of Individual *sod* Genes Results in Mild Compensatory Upregulation of *sod* mRNA. A) *sod* mRNA levels for each of the five *sod* genes was examined in the *sod* deletion mutants by quantitative real-time RT-PCR. While compensatory upregulation was observed in *sod-2*, *sod-3* and *sod-5* mutant worms, the magnitude was small, generally 2-fold or less. No *sod* mRNA upregulation in *sod-1* or *sod-4* mutant worms. B) Examination of SOD-1 and SOD-2 protein levels by Western blotting reveals no SOD-1 expression in *sod-1* mutant worms and no SOD-2 expression in *sod-2* mutant worms. C) Quantification of SOD protein levels reveals no significant upregulation of SOD-1 or SOD-2 proteins in any of the *sod* deletion mutants. * p<0.05.

In order to confirm the absence of individual *sod* expression in the deletion strains, we assessed the levels of each of the five *sod* mRNAs by qRT-PCR (quantitative real time reverse transcription polymerase chain reaction). Importantly, we also sought to determine whether the deletion of individual *sod* genes is compensated for by the upregulation of other *sod* genes. In all strains, the deletion mutation resulted in decreased levels of the corresponding mRNA (in most cases no mRNA was detected) (Figure 2A). In both *sod-2* mutant strains, *sod-1*, *sod-3* and *sod-4* mRNAs were significantly elevated and there was a trend towards increased expression of *sod-5* (Figure 2A). Similarly, *sod-3* mutant worms showed increased expression of *sod-1* and *sod-4* mRNA and a trend towards increased expression of *sod-2* and *sod-5* (Figure 2A). One of two *sod-5* mutants showed significantly increased expression of *sod-1* mRNA, while *sod-1* and *sod-4* mutant worms showed no significant changes in mRNA levels of the other four *sod* mRNAs (Figure 2A). Overall, we observed some compensatory upregulation of other *sod* mRNAs in the *sod* deletion strains but the degree of upregulation was small, generally 2-fold or less. The fact that *sod-3* mutant worms showed a similar upregulation of *sod* mRNA as *sod-2* mutant worms suggests that the lifespan extension in the *sod-2* mutants does not result from the observed upregulation of *sod* mRNA.

Since a compensatory increase in SOD expression could also occur at the translational level, we examined the level of SOD-1 and SOD-2 protein in the *sod* deletion mutants (antibodies to the other SOD proteins are currently not available). We observed no SOD-1 protein in *sod-1* deletion mutants or SOD-2 protein in *sod-2* deletion mutants (Figure 2B). As with mRNA expression we did not observe a dramatic upregulation of SOD-1 or SOD-2 in any of the *sod* deletion mutants (Figure 2C).

### Increased Sensitivity to Oxidative Stress Does Not Decrease Lifespan in *sod-sod* Double Mutants

Although the magnitude of compensatory upregulation of other *sod* genes, when present, was small, it is possible that this could have accounted for the normal or extended lifespan we observed in the *sod* single deletion mutants. To investigate this possibility, we sought to determine whether elimination of a second *sod* gene would shorten the lifespan of the *sod* single deletion mutants. Accordingly, we generated a panel a *sod-sod* double mutants consisting of all of the double mutants for *sod-1* and *sod-2*, since these are the major contributors to SOD activity in the cytosol and mitochondria respectively, and *sod-3*; *sod-5* (this mutant lacks both of the “extra” *sod* genes found in *C. elegans*).

**Figure 3:**
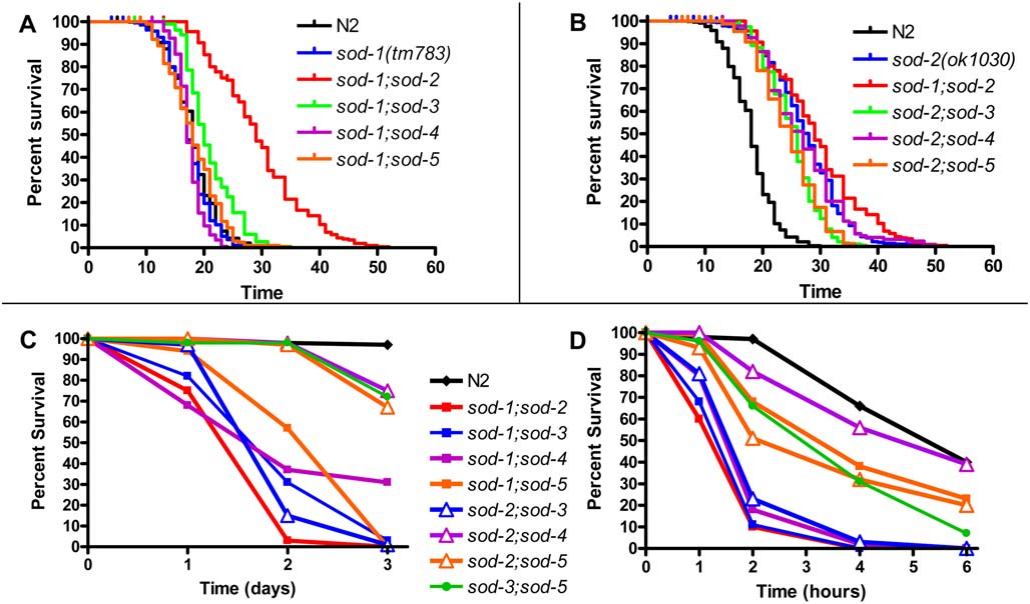
*sod-sod* Double Deletion Mutants Do Not Exhibit Decreased Lifespan Despite Increased Sensitivity to Oxidative Stress. A) None of the *sod-1* double deletion mutants showed a decreased lifespan compared to wild-type or *sod-1* mutant worms. *sod-1;sod-2* mutant worms show extended longevity. B) All of the *sod-2* double deletion mutants exhibited extended lifespan compared to wild-type worms. C) Despite exhibiting a normal or extended lifespan, all of the *sod-1* double deletion mutants as well as *sod-2;sod-3* mutant worms were very sensitive to paraquat-induced oxidative stress. D) As with the paraquat assay, *sod-1* double deletion mutants and *sod-2;sod-3* mutant worms showed the greatest sensitivity to juglone-induced oxidative stress. Overall, *sod-sod* double deletion mutants show increased sensitivity to oxidative stress but exhibit a normal or extended lifespan.

Examining the lifespan of *sod-1* double deletion mutants revealed that deletion of *sod-3*, *sod-4* or *sod-5* did not shorten the lifespan of *sod-1* mutant worms (Figure 3A). In contrast, *sod-1;sod-2* mutant worms lived significantly longer than wild-type N2 worms (Figure 3A). Among the *sod-2* double deletion mutants, all of the worms maintained the extended lifespan seen in *sod-2* single deletion mutants indicating that in no case is the upregulation of another *sod* gene entirely responsible for the long life observed in *sod-2* mutant worms (Figure 3B). Finally, we found that *sod-3;sod-5* mutant worms had a similar lifespan to wild-type worms (not shown).

Next, we examined the sensitivity to oxidative stress among the *sod-sod* double mutants using both paraquat and juglone. Examining the survival of one day old adult worms on 4 mM paraquat plates, we found that all of the *sod-1* double mutants, including the long-lived *sod-1;sod-2* mutant worms, had decreased survival compared to N2 worms (Figure 3C). Among the *sod-2* double mutants, *sod-2;sod-3* mutant worms were hypersensitive to paraquat, while *sod-2;sod-4* and *sod-2;sod-5* mutant worms appeared to be only mildly more sensitive than N2 worms (Figure 3C).

A similar pattern of sensitivity to oxidative stress was observed on juglone plates. All of the *sod-1* double mutants as well as *sod-2;sod-3* mutant worms were more sensitive to juglone than N2 worms (Figure 3D). There was also a trend towards decreased survival in the remaining double mutant strains (Figure 3D). Overall, the *sod-sod* double mutants showed increased sensitivity to oxidative stress but normal or extended longevity. Thus, we did not observe any correlation between sensitivity to oxidative stress and lifespan.

We also examined *sod* mRNA expression levels in the *sod-sod* double mutant worms (Figure S4). As with the *sod* single deletion mutants, we observed some compensatory upregulation of other *sod* genes but the magnitude of this increase was small and failed to rescue the observed increase in sensitivity to oxidative stress.

### Mutants With Deletions in Three *sod* Genes Exhibit Normal or Extended Lifespan

**Figure 4:**
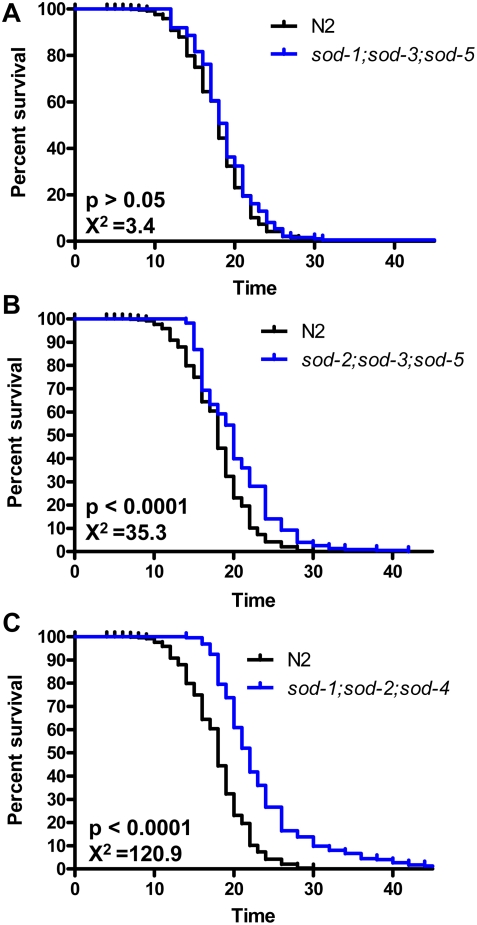
Worms Lacking Combinations of Three *sod* Genes Show Normal or Extended Lifespan. To model *sod-1* and *sod-2* knockouts in species which have only three *sod* genes, *sod-1;sod-3;sod-5* (A) and *sod-2;sod-3;sod-5* (B) mutant worms were generated respectively. *sod-1;sod-2;sod-4* (C) mutant worms which lack the primary cytoplasmic, mitochondrial and extracellular *sod* genes were also generated. Surprisingly, none of the *sod* triple deletion mutants showed decreased lifespan compared to wild-type worms and the two triple mutants bearing the *sod-2* allele lived significantly longer than wild-type. Thus, *C. elegans* is able to compensate for the loss of multiple *sod* genes to achieve a normal or extended lifespan.

Based on our finding that even the elimination of two *sod* genes together does not shorten the lifespan of *C. elegans*, we assayed lifespan in a selection of *sod* triple mutants. To eliminate the possibility that the reason why *sod-1* and *sod-2* mutants of *C. elegans* do not show decreased lifespan is because *C. elegans* has duplicate SODs in the cytoplasm and mitochondria, we generated *sod-1;sod-3;sod-5* and *sod-2;sod-3;sod-5* triple mutants to model *sod-1* and *sod-2* knockouts in species with only three *sod* genes. We also generated *sod-1;sod-2;sod-4* worms which lack the primary cytoplasmic, mitochondrial and extracellular *sod* genes. Examination of worm lifespan revealed that *sod-1;sod-3;sod-5* mutant worms live as long as wild-type worms while both *sod-2;sod-3;sod-5* and *sod-1;sod-2;sod-4* mutant worms live significantly longer than wild-type (Figure 4A–C). This clearly indicates that the normal lifespan observed in *sod-1* worms does not result from the overlapping expression of *sod-3* in the mitochondria or *sod-5* in the cytoplasm. Since *sod-2;sod-3;sod-5* triple mutant worms do not survive as long as *sod-2* single deletion mutants, it is possible that the mild upregulation of *sod-3* and *sod-5* may contribute to the increased lifespan of *sod-2* mutant worms. However, the fact that similar upregulation of *sod* mRNA in *sod-3* mutant worms does not result in extension of lifespan and that upregulation of *sod-3* and *sod-5* in *sod-2* mutant worms is insufficient to prevent increased levels of oxidative damage (see below) suggests that other mechanisms are involved in the long life of *sod-2* mutant worms.

### Interaction of *sod-2* Deletion With Lifespan Extending Mechanisms in Known Genetic Pathways

**Figure 5:**
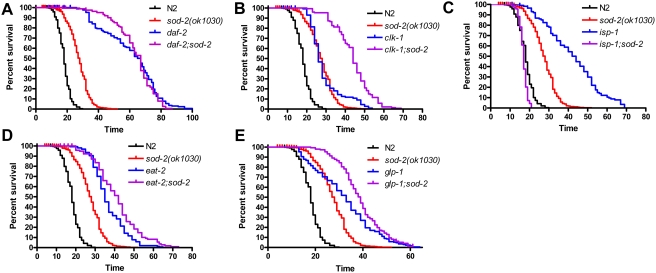
*sod-2* Deletion Alters the Lifespan of Extended Longevity Mitochondrial Mutants. To gain insight into the mechanism of lifespan extension in *sod-2* mutant worms, we examined the effect of *sod-2* deletion on other genes known to increase longevity. A) *sod-2* deletion did not affect lifespan in *daf-2* worms (insulin/IGF1 signaling). B) *sod-2* deletion markedly increases lifespan in *clk-1* worms (decreased mitochondrial function). C) *sod-2* deletion shortens lifespan in *isp-1* worms (decreased mitochondrial function) such that *isp-1;sod-2* worms do not survive as long as wild-type worms. D) *sod-2* deletion moderately extends mean and maximum lifespan in *eat-2* worms (dietary restriction). E) *sod-2* deletion extends mean but not maximum lifespan of *glp-1* worms (germ-line ablation). Thus, *sod-2* deletion has the greatest impact on the lifespan of *clk-1* and *isp-1* worms, which extend lifespan by decreasing mitochondrial function.

In order to investigate possible mechanisms of lifespan extension in *sod-2* mutant worms, we generated double mutants with genes known to extend lifespan which are representative of different lifespan extending mechanisms including *daf-2* (insulin/IGF signaling)[29], *clk-1* (decreased mitochondrial function)[30], *isp-1* (decreased mitochondrial function)[25], *eat-2* (dietary restriction)[31] and *glp-1* (germ-line ablation)[32]. Deletion of *sod-2* did not extend the lifespan of *daf-2* worms (Figure 5A). *clk-1* worms showed a marked extension of lifespan when *sod-2* was deleted (Figure 5B). In contrast, *sod-2* deletion greatly shortened the lifespan of *isp-1* worms such that *isp-1;sod-2* worms had a shorter lifespan than wild-type N2 worms (Figure 5C). *eat-2;sod-2* mutant worms showed a small increase in lifespan compared to *eat-2* worms (Figure 5D). Finally, deletion of *sod-2* in *glp-1* worms resulted in a modest increase of mean but not maximum lifespan (Figure 5E). Overall, we found that *sod-2* deletion had the greatest impact on the lifespan of mutants which exhibit extended longevity as a result of alterations in mitochondrial function.

### *sod-2* Mutant Worms Exhibit Phenotypes Characteristic of Extended Longevity Mitochondrial Mutants

Based on our finding that *sod-2* deletion interacts with long-lived mutants with altered mitochondrial function and the fact that SOD-2 is localized to the mitochondria, we hypothesized that deletion of *sod-2* extends lifespan by decreasing mitochondrial function. In *C. elegans* a number of genes have been identified that affect mitochondrial function and at the same time increase lifespan [25],[30],[33],[34]. Although these genes do not necessarily interact and the exact mechanism of lifespan extension is unclear, these mutants are generally grouped together since it is believed that the alteration of mitochondrial function is the key to their long life. In addition to impaired mitochondrial function and extended longevity, these mutants, sometimes referred to as Mit mutants, are characterized by slow development, slow defecation rate and decreased brood size. Accordingly, we quantified the development, brood size and defecation rate of *sod-2* mutant worms and compared this with two prototypes of this class of mutants - *clk-1* and *isp-1* worms [25],[30],[35]. We also examined *clk-1;sod-2* and *isp-1;sod-2* double mutants to determine if the loss of *sod-2* enhanced the phenotypes observed in *clk-1* and *isp-1* worms.

**Figure 6:**
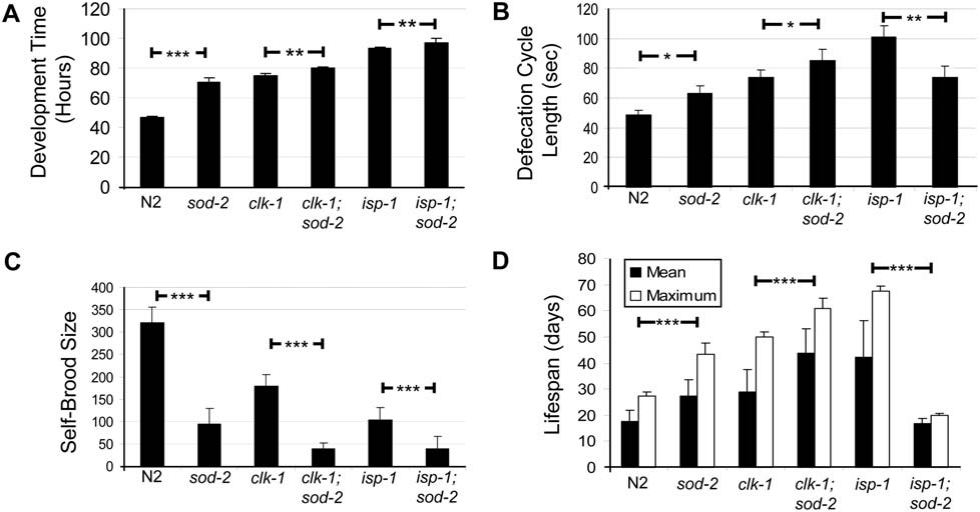
*sod-2* Mutant Worms Exhibit the Hallmark Features of Extended Longevity Mitochondrial Mutants in *C. elegans*. Similar to extended longevity mitochondrial mutants such as *clk-1* and *isp-1*, *sod-2* mutant worms show slow post-embryonic development (A), slow defecation rate (B), decreased self-brood size (C) and long life (D). In *clk-1* worms, the deletion of *sod-2* slows development, slows defecation rate, decreases brood size and increases lifespan. In *isp-1* worms, *sod-2* deletion slows development, increases defecation rate, decreases brood size and decreases lifespan. *p<0.05, **p<0.01, ***p<0.001.

Examination of post-embryonic development (PED) revealed that *sod-2*, *clk-1* and *isp-1* worms all developed slower than wild-type worms (Figure 6A). On a *clk-1* and *isp-1* background, *sod-2* deletion resulted in further increase in PED time (Figure 6A). Examination of defecation cycle length revealed a slow rate of defecation in *sod-2*, *clk-1* and *isp-1* worms compared to wild-type N2 worms (Figure 6B). Deletion of *sod-2* had opposite effects on defecation cycle length in *clk-1* and *isp-1* worms. *sod-2* deletion further lengthened the defecation cycle of *clk-1* worms but shortened the defecation cycle length of *isp-1* worms (Figure 6B). Self-brood size was decreased in *sod-2*, *clk-1* and *isp-1* worms and deletion of *sod-2* further decreased the brood size in *clk-1* and *isp-1* worms (Figure 6C). Finally, a comparison of lifespan between these strains revealed that *isp-1;sod-2* worms were short-lived, *sod-2* and *clk-1* worms were long-lived and *clk-1;sod-2* and *isp-1* worms were very long-lived (Figure 6D). Clearly, *sod-2* deletion mutants exhibit the key characteristics of extended longevity mitochondrial mutants and modulate these phenotypes in *clk-1* and *isp-1* worms.

### Oxygen Consumption Is Decreased in *sod-2* Mutant Worms

**Figure 7:**
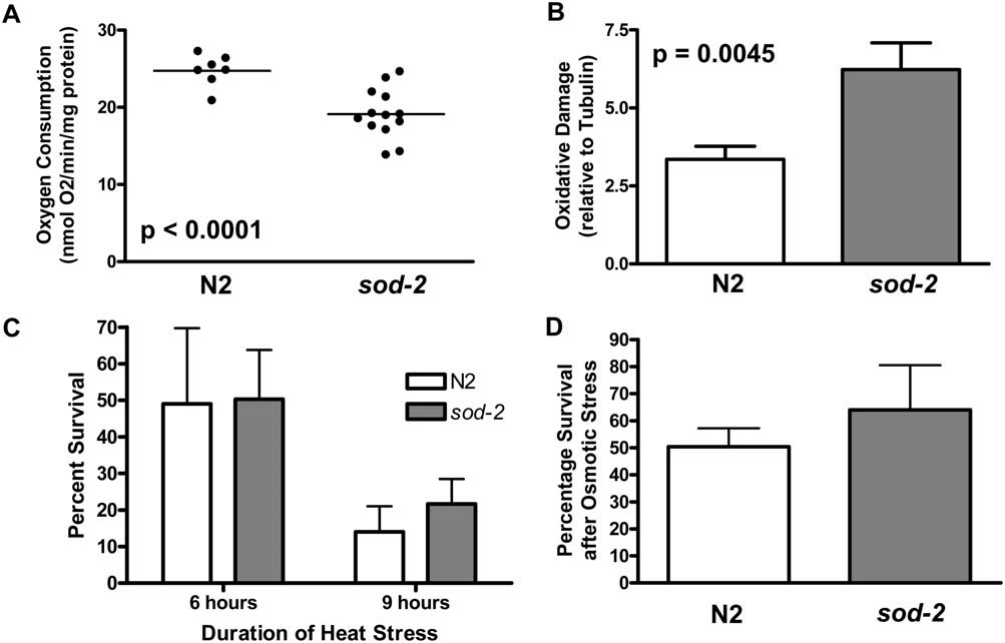
*sod-2* Mutant Worms Show Decreased Oxygen Consumption and Increased Oxidative Damage But Normal Tolerance to Heat and Osmotic Stress. A) In order to assess mitochondrial function in *sod-2* mutant worms, whole worm oxygen consumption was measured and compared to wild-type N2 controls. Oxygen consumption was significantly decreased in *sod-2* mutant worms compared to wild-type worms. B) Examination of carbonylated proteins by Oxyblot reveals increased oxidative damage in *sod-2* mutant worms compared to wild-type worms (N = 10 N2, 8 *sod-2*). Nonetheless, *sod-2* mutant worms survive both heat stress (C) and osmotic stress (D) as well as wild-type worms (stress results represent three independent trials).

The phenotypic similarity of *sod-2* mutant worms to extended longevity mitochondrial mutants as well as the ability of *sod-2* deletion to alter these characteristic phenotypes in *clk-1* and *isp-1* worms suggests that *sod-2* extends lifespan through a similar mechanism. Based on this hypothesis, we would predict that mitochondrial function would be altered in *sod-2* mutant worms. To assess this, we measured whole worm oxygen consumption, which has previously been shown to be decreased in both *clk-1* and *isp-1* worms [16],[25]. We found that oxygen consumption in one day old adult worms was significantly decreased in *sod-2* mutant worms compared to wild-type worms (Figure 7A).

### Oxidative Damage Is Increased in *sod-2* Mutant Worms

We have previously reported that both *clk-1* and *isp-1* worms exhibit decreased levels of oxidatively damaged proteins [16]. Here, we find that *sod-2* worms are hypersensitive to oxidative stress, suggesting that there may be an increase in oxidatively damaged proteins in these worms. To determine the level of oxidative damage in *sod-2* mutant worms, we quantified carbonylated proteins in *sod-2* mutant worms and wild-type worms. We found that *sod-2* mutant worms had significantly more oxidative damage than wild-type worms (Figure 7B).

### *sod-2* Mutant Worms Are Not Hypersensitive to Heat or Osmotic Stress

In order to determine whether the sensitivity of *sod-2* mutant worms to paraquat and juglone was the result of a specific sensitivity to oxidative stress or a sign of general weakness, we examined the ability of *sod-2* worms to withstand heat stress [36] or osmotic stress [37]. After exposure to 35 degree Celsius heat stress for a period of 6 hours or 9 hours, we found that *sod-2* mutant worms survived as well as wild-type worms (Figure 7C). Similarly, exposing *sod-2* mutant worms to osmotic stress on 500 mM NaCl NGM plates for 20 hours revealed that *sod-2* mutant worms survive osmotic stress as well as wild-type N2 worms (Figure 7D). Combined these results suggest that the sensitivity of *sod-2* mutant worms to oxidative stress is a specific sensitivity resulting from their decreased ability to detoxifying ROS.

## Discussion

In this paper we test the oxidative stress theory of aging in *C. elegans*. Examination of lifespan and sensitivity to oxidative stress in mutants with deletions in one, two or three *sod* genes reveals normal or extended lifespan in worms with markedly increased sensitivity to oxidative stress. In contrast to observations in other species, deletion of *sod-2* results in long life despite increased levels of oxidative damage. The lifespan extending mechanism of *sod-2* interacts most strongly with that of *clk-1* and *isp-1* mutations, which extend lifespan by decreasing mitochondrial function. The phenotypic similarity of *sod-2* mutant worms with these mitochondrial mutants, the mitochondrial localization of SOD-2 and the decreased oxygen consumption in *sod-2* mutant worms suggest that *sod-2* deletion extends lifespan through alterations in mitochondrial function.

### Oxidative Stress Theory of Aging

Since its origins in 1956, the oxidative stress theory of aging has been extensively tested in multiple organisms both by observing variations in natural populations and through genetic intervention [1]–[3]. Thus far there have been many experiments that support this theory, but also experiments which challenge the notion that molecular damage from ROS leads to aging (reviewed in [38]).

In this paper, we find that the effect of *sod* deletion on lifespan in *C. elegans* is unique from other organisms. In concordance with the oxidative stress theory of aging, yeast, flies and mice lacking either cytoplasmic or mitochondrial SOD show either decreased lifespan or lethality (in the case of *Sod2* knockout mice) [5], [7], [9], [10], [12]–[14] while mice lacking extracellular SOD live as long as wild-type mice [15]. Here, we demonstrate that none of the *sod* deletion mutants in *C. elegans* show decreased lifespan. One possible explanation for this discrepancy is the fact that *C. elegans* has five *sod* genes, rather than three, including two cytoplasmic SODs and two mitochondrial SODs. To eliminate this explanation, we show that the lifespan of *sod-1;sod-3;sod-5* and *sod-2;sod-3;sod-5* triple mutants, which model *sod-1* and *sod-2* deficient organisms in species with only three *sod* genes, is not decreased.

Another possible explanation for why *C. elegans sod* mutants exhibit a normal lifespan would be compensatory upregulation of other *sod* genes. In support of this hypothesis, we observed *sod* mRNA upregulation in *sod-2* and *sod-3* mutant worms as well as one of two *sod-5* mutants. However, the magnitude of this upregulation was small (2-fold or less) and we observed no significant *sod* upregulation in *sod-1* or *sod-4* mutant worms which also exhibit a normal lifespan. These results are in general agreement with studies of *Sod* knockouts in flies and mice where either no change in other SOD activity is reported [12], [15], [39]–[41] or changes with magnitudes of less than 50% [10],[14],[42].

Although the compensatory upregulation of other *sod* genes was small in magnitude and not present in all *sod* deletion mutants, it is possible that this small increase contributed to the normal or extended lifespans observed in these strains. It is also possible that changes in SOD protein levels or activity contributed to the preservation of lifespan in these strains. To investigate these possibilities, we used the genetic approach of generating *sod* double and triple mutants. The loss of an additional *sod* gene did not decrease the lifespan in *sod-1* mutant worms, nor did it revert the lifespan of *sod-2* mutant worms to wild-type. This indicates that the increased expression or activity of any single other *sod* gene is not responsible for the normal lifespan observed in *sod-1* mutant worms or extended lifespan observed in *sod-2* mutant worms. Similarly, all of the *sod* triple mutants were able to live at least as long as wild-type worms. The lack of lifespan shortening in worms with multiple *sod* genes deleted is in concordance with studies in mice where the loss of extracellular SOD [43] or the loss of glutathione peroxidase (another ROS detoxifying enzyme) and one copy of *Sod2* [38] does not further decrease lifespan in *Sod1* knockout mice. The lack of additive effects between different compartments can be explained by the inability of superoxide to cross biological membranes [44],[45]. A compartment specific effect of genes involved in ROS detoxification on lifespan has also been observed in *C. elegans* with genes encoding catalase, where deletion of peroxisomal catalase (*ctl-2*) results in decreased lifespan while deletion of cytoplasmic catalase (*ctl-1*) has no effect on lifespan [46].

A comparison of our results for lifespan and sensitivity to oxidative stress reveals no correlation. None of the *sod* single or double mutants exhibited a shortened lifespan despite many strains showing markedly increased sensitivity to oxidative stress. Most strikingly, *sod-1;sod-2* mutants show the highest sensitivity to oxidative stress in combination with the longest lifespan. Similarly, our laboratory has recently shown that decreasing levels of *sod-1* or *sod-2* by RNAi increases paraquat sensitivity and oxidative damage to proteins in N2 as well as in multiple long-lived strains (*daf-2*, *clk-1* and *isp-1*) yet does not decrease lifespan in these strains [16]. Initial experiments examining sensitivity to oxidative stress in long-lived worms indicated that increased resistance to oxidative stress occurs with increased lifespan [24],[47],[48]. It was also found that when longevity was selected for in flies, increased longevity was accompanied by resistance to oxidative stress [49]. More recently, in the reverse experiment examining the lifespan of worms that were resistant to paraquat-induced oxidative stress, it was found that only 84 of 608 RNAi treatments that increased stress resistance also increased lifespan [50]. Similarly, examination of the relationship between paraquat resistance and lifespan in 138 lines of flies revealed only a weak positive correlation [51]. In mice, *Sod1* knockouts show increased sensitivity to oxidative stress and decreased lifespan [10],[39], mice heterozygous for the targeted inactivation of *Sod2* showed a normal lifespan despite increased oxidative damage [52], while *Sod3* knockout mice show increased sensitivity to oxidative stress and a normal lifespan [15]. Combined with our results, it appears that the correlation between sensitivity to oxidative stress and lifespan is weak at best.

### Extended Longevity Resulting From *sod-2* Deletion Is Unique to *C. elegans*

SOD2 is the primary, and normally sole, SOD present in the mitochondrial matrix. Since the mitochondria is a major source of superoxide within the cell and superoxide is not able to pass through membranes[44],[45], SOD2 may be the most critical SOD within the cell for decreasing superoxide-induced damage. This conclusion is supported by findings that decreasing or eliminating SOD2 expression affects lifespan more than elimination of SOD1 or the extracellular SOD. In flies, eliminating SOD1 reduces lifespan from about 60 days to 11.8 days [9] while eliminating SOD2 decreases lifespan to less than 1 day [12]. Similarly, *Sod1* knockout mice show a 30% decrease in lifespan living an average of 20.8 months [10], while *Sod2* knockout mice exhibit either peritnatal or neonatal lethality [13],[14].

In contrast to what is observed in other species, we find that *sod-2* deletion in *C. elegans* results in extended lifespan. While these worms show small but significant increases in *sod-1*, *sod-3* and *sod-4* mRNA expression, deleting *sod-1*, *sod-3* or *sod-4* in *sod-2* mutant worms does not revert their lifespan to wild-type suggesting that this upregulation of *sod* expression is not responsible for the lifespan increase in *sod-2* mutant worms. Our observation of decreased lifespan in *sod-2;sod-3;sod-5* mutant worms compared to *sod-2* mutant worms suggests the possibility that upregulation of *sod-3* and *sod-5* partially contributes to the extended lifespan observed in *sod-2* mutant worms. However, the fact that we observe similar upregulation of other *sod* genes in *sod-3* mutant worms without the lifespan extension supports the conclusion that the mild compensatory upregulation of other *sod* genes is not responsible for the long life of *sod-2* mutants. Furthermore, the fact that *sod-2* mutant worms show increased oxidative damage indicates that the upregulation of *sod-3* and *sod-5* is not sufficient to reduce mitochondrial oxidative stress in *sod-2* mutant worms.

### Increased Longevity through Alteration of Mitochondrial Function

To gain insight into the mechanism of lifespan extension in *sod-2* mutant worms, we examined the effect of *sod-2* deletion on other mutants with extended longevity. *sod-2* deletion did not extend lifespan in *daf-2* worms, which extend lifespan through the insulin-IGF1 pathway [29] but did result in a modest extension of lifespan in *eat-2* worms, which extend lifespan through caloric restriction [31] and *glp-1* worms, which extend lifespan through the germ-line ablation [32]. In *clk-1* worms, which extend lifespan by decreasing mitochondrial function [30],[53], deletion of *sod-2* resulted in a 15 day increase in mean lifespan. In contrast, *sod-2* deletion decreased the lifespan of *isp-1* worms by 25 days despite the fact that *isp-1* also extends lifespan through via a decrease in mitochondrial function [25].

The clear interaction of *sod-2* deletion with mutants that extend lifespan through alterations in mitochondrial function suggested the possibility that *sod-2* also increases longevity through a similar mechanism. In *C. elegans* a number of mutants have been identified by genetic deletion or RNAi that affect mitochondrial function and extend lifespan [25],[30],[33],[34],[54]. In addition to decreased mitochondrial function and extended lifespan, the group of mitochondrial mutants also share a number of characteristic phenotypes such as slow rate of development, slow rate of defecation and decreased brood size [25],[35],[55]. Phenotypic characterization of *sod-2* mutant worms demonstrates that *sod-2* mutants exhibit all of the phenotypes of the extended longevity mitochondrial mutants including slow development, slow defecation rate, decreased brood size, decreased mitochondrial function and increased lifespan.

While we have previously shown that *clk-1* and *isp-1* worms have decreased levels of oxidatively damaged proteins [16], here we find that *sod-2* mutant worms exhibit an increase in oxidative damage. The fact that all three strains have a long lifespan suggests that both high and low levels of oxidative damage are compatible with long life. Moreover, the fact that oxidative damage in *clk-1* and *isp-1* worms can be increased to a level that is significantly greater than wild-type worms without diminishing the long life of these two strains suggests that the low levels of oxidative damage in *clk-1* and *isp-1* worms does not contribute to their extended longevity [16].

In addition to those genes which impair mitochondrial function and increase lifespan, there are at least two mutations, *mev-1* [56] and *gas-1* [57], which decrease mitochondrial function and decrease lifespan. While it is currently uncertain why these mutations have a different effect on lifespan compared to the extended longevity mitochondrial mutants, it appears that there are at least two ways in which decreasing mitochondrial function can lead to decreased lifespan. First, the severity of the mutation can be incompatible with long life. This has recently been demonstrated using an RNAi dilution series against genes involved in mitochondrial function [55]. These authors find that RNAi against the same gene can increase lifespan at low concentration (i.e. mildly inhibited mitochondrial function) and decrease lifespan at high concentration (i.e. severely inhibited mitochondrial function). In our work, we hypothesize that *isp-1;sod-2* worms are another example whereby the overall mitochondrial function in the double mutant worm is severely affected leading to a shortened lifespan. Second, the decreased lifespan can be the result of the way in which mitochondrial function is altered. For example, RNAi targeted against any of the four subunits of electron transport chain complex II results in decreased lifespan [58] while RNAi targeted against proteins in any other complex of the electron transport chain can result in increased lifespan [33]. Furthermore, recent work examining the effect of an RNAi dilution series against *mev-1* indicates that it is not the severity of this mutation that prevents it from extending lifespan, since *mev-1* RNAi failed to increase the lifespan of wild-type worms at any concentration [55].

### Modulation of *clk-1* and *isp-1* Phenotypes by Deletion of *sod-2*

**Figure 8:**
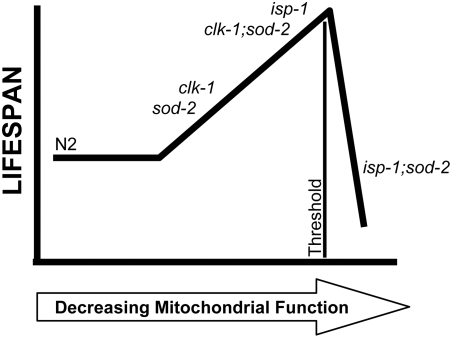
Proposed Model Relating Mitochondrial Function and Lifespan in *sod-2* Mutant Worms and *sod-2* Double Mutants. N2 exhibits normal mitochondrial function and a normal lifespan. In *sod-2* and *clk-1* worms, mitochondrial function is decreased leading to compensatory changes which increase lifespan. Deleting *sod-2* in *clk-1* worms further decreases mitochondrial function towards that of *isp-1* worms with a coincident increase in lifespan. Deleting *sod-2* in *isp-1* worms decreases mitochondrial function past a threshold after which the organism is no longer able to compensate for the degree of mitochondrial impairment and lifespan decreases.

Examination of *clk-1;sod-2* double mutants shows that *sod-2* deletion enhances all of the mitochondrial mutant phenotypes of *clk-1* worms. However, a different pattern is observed with *isp-1* worms, where *sod-2* deletion further slows development and decreases brood size but quickens defecation towards wild-type and decreases lifespan below wild-type N2 worms. We propose that the reason for the different effects of *sod-2* deletion on *clk-1* and *isp-1* worms results from differences in the initial degree of mitochondrial function. Based on previous measurements of oxygen consumption, respiration is only mildly impaired in *clk-1* worms [16],[53] while it is more than 50% reduced in *isp-1* worms [25]. By comparing the other phenotypes of N2, *clk-1* and *isp-1* worms it can be seen that as mitochondrial function decreases, development time gets longer, defecation gets slower, self brood size decreases and lifespan increases. However, according to mitochondrial threshold theory, once a certain threshold of mitochondrial dysfunction is reached, the cell is no longer able to compensate and lifespan decreases [59]. This theory was recently explored in *C. elegans* through the use of an RNAi dilution series to show that progressively decreasing mitochondrial function resulted in increased lifespan only until a certain threshold after which lifespan began to decrease [55]. Based on these findings, we propose a model in which the shortened lifespan that we observe in *isp-1;sod-2* worms results from the *sod-2* deletion pushing mitochondrial function past the threshold at which the organism is able to compensate for the lost mitochondrial function and accordingly lifespan is decreased (Figure 8). Similarly, we propose that the increased lifespan in *clk-1;sod-2* worms results from the *sod-2* deletion reducing the mitochondrial function to a level similar to *isp-1* worms. In line with our demonstration of decreased oxygen consumption in *sod-2* mutant worms, deletion of *sod-2* has also been shown to decrease mitochondrial function in mouse models [60]–[62].

Although the first of the extended longevity mitochondrial mutants was identified more than a decade ago [30],[35],[54], the precise mechanism by which these mutants extend lifespan is still unresolved. Nonetheless, a number of potential mechanisms have been suggested [63]. Future studies will need to more precisely define how mitochondrial mutants, such as *sod-2*, extend lifespan and to determine how *C. elegans* is able to cope with reduced SOD activity. It will be particularly interesting to examine the interaction between SODs and other proteins involved in ROS detoxification (catalases, peroxidases, thioredoxins, peroxiredoxins) in order to obtain a more complete understanding of the relationship between oxidative stress and lifespan.

## Materials and Methods

### Strains

The following strains were used in these experiments: N2 (wild-type), *sod-1(tm776)*, *sod-1(tm783)*, *sod-2(gk257)*, *sod-2(ok1030)*, *sod-3(tm760)*, *sod-4(gk101)*, *sod-5(tm1146)*, *sod-5(tm1246)*, *clk-1(qm30)*, *eat-2(ad1116)*, *daf-2(e1370)*, *isp-1(qm150)*, *glp-1(e2141)*. Strains obtained from external sources were outcrossed with our N2 worms for 5-10 generations. For these experiments the following double and triple mutant strains were generated: *sod-1(tm783);sod-2(ok1030)*, *sod-1(tm783);sod-3(tm760)*, *sod-1(tm783);sod-4(gk101)*, *sod-1(tm783);sod-5(tm1246)*, *sod-2(ok1030);sod-3(tm760)*, *sod-2(ok1030);sod-4(gk101)*, *sod-2(ok1030);sod-5(tm1246)*, *sod-3(tm760);sod-5(tm1246)*, *clk-1(qm30);sod-2(ok1030)*, *eat-2(ad1116);sod-2(ok1030)*, *daf-2(e1370);sod-2(ok1030)*, *isp-1(qm150);sod-2(ok1030)*, *glp-1(e2141);sod-2(ok1030)*, *sod-1(tm783);sod-2(ok1030);sod-4(gk101)*, *sod-1(tm783);sod-3(tm760);sod-5(tm1246)*, and *sod-2(ok1030);sod-3(tm760);sod-5(tm1246)*. All of the *sod* deletions were confirmed by PCR. All strains were maintained at 20°C.

### Lifespan Analysis

Lifespan studies were completed at 20°C with a minimum of 3 independent trials and an initial number of 80 worms per strain per trial. Initial lifespan assays for *sod* single deletion mutants, *sod-sod* double deletion mutants and *sod-2* double mutants with genes in known pathways of lifespan extension were completed on normal NGM plates. As some *sod* double mutant strains bagged extensively subsequent lifespan studies were completed on plates containing 100 µM FUDR (Sigma). Results obtained on NGM plates were all repeated and confirmed on FUDR plates. Survival plots shown represent pooled data from multiple trials on FUDR plates. For *glp-1* and *glp-1;sod-2* lifespan analyses worms were grown at 25°C and then transferred to 20°C at adulthood.

### Paraquat and Juglone Sensitivity

Paraquat and juglone sensitivity assays were completed in triplicate with 30–40 worms per strain per trial at 20°C. To assay paraquat sensitivity, 7 day old adult worms were transferred to plates containing 4 mM paraquat (Sigma) and survival was monitored daily. Initially, paraquat assays were performed on 1 day old adult worms. However, by day 3 of adulthood, paraquat causes most of the worms to have internal hatching of progeny (bagging) such that more worms die of this than of paraquat toxicity.

Juglone sensitivity was assessed in 1 day old adult worms on plates containing 240 µM juglone (Sigma). For this assay, plates were made fresh on the day of the assay as the toxicity of juglone decreases rapidly over time. Survival was monitored for 6 to 10 hours. To assess the ability of worms to develop under oxidative stress, a minimum of 40 eggs were placed on plates containing 0.2 mM paraquat and seeded with OP50 bacteria.

### Quantitative Real-Time RT-PCR

RNA was isolated from young adult worms using TRIZOL reagent (Invitrogen). Subsequently, 1 µg of RNA was converted to cDNA using the Quantitect Reverse Transcription kit (Qiagen). 1 µl of the resulting cDNA preparation was used for quantitative real-time PCR using the Quantitect SYBR Green PCR kit and a Biorad iCycler RT-PCR machine. Primer sequences for *sod* mRNAs were previously validated [64]. A combination of three control primer sets (*cdc-42*, *pmp-3* and *Y45F10D.4*) were used as has been previously described [65]. Results represent the average of three independent biological samples, each of which was amplified in triplicate.

### Post-Embryonic Development

Eggs were collected and allowed to hatch over a period of 3 hours. After 3 hours, L1 worms were transferred to a new plate and monitored for development to an adult worm. Results are the average of at least three independent trials with 20 worms per trial.

### Defecation

Defecation cycle length in young adult worms was measured as the average time between consecutive pBoc contractions. Results represent a minimum of 3 trials with 10 worms per trial.

### Self-Brood Size

To determine the average number of progeny produced by each strain, L4 worms were placed on individual NGM plates. Worms were transferred daily until egg laying ceased and the total number of live progeny produced was counted.

### Oxygen Consumption

Gravid adult worms were collected from five to ten 100 mm NGM plates and bleached to recover eggs. Eggs were allowed to hatch in M9 buffer over a period of 5 days before L1 worms were transferred to NGM plates. At adulthood worms were collected in M9 buffer, washed free of bacteria and oxygen consumption was measured using a Clark electrode for a 10 minute period. Subsequently worms were pelleted and frozen for protein quantification. Proteins were quantified using a bicinchonic acid protein assay kit (Thermo Scientific) according to the manufacturer's protocol.

### Western Blotting and Detection of Carbonylated Proteins

Western blotting for SOD proteins was completed as described previously [16]. Antibody dilutions were as follows: SOD-1 (1∶1000), SOD-2 (1∶1000), tubulin (1∶10,000). Levels of protein were compared in three independent samples of one day old adult worms. Oxidative damage was assessed using an Oxyblot assay kit (Millipore) to detect carbonylated proteins as previously described [16]. In this assay carbonyl groups are derivatized to 2,4-dinitrophenylhydrazone (DNP-hydrazone) which can then be detected by western blotting with a DNP specific antibody. The Oxyblot assay was completed according to the manufacturer's protocol using 10 samples of N2 worms and 8 samples of *sod-2* mutant worms (Millipore). 9 µg of protein lysate was loaded in each lane. Quantification of carbonylated proteins was achieved by taking the ratio of DNP staining to tubulin staining.

### Heat Stress and Osmotic Stress

Heat stress experiments were based on previously developed protocols [36]. Briefly, young adult worms on NGM plates were incubated at 35 degrees Celsius for a period of 6 or 9 hours. Worms were then transferred to a 20 degree Celsius incubator. Two days later the percentage of worms surviving was determined. Osmotic stress experiments were also done according to previously developed protocols [37]. Young adult worms were transferred to NGM plates containing 500 mM NaCl. After 20 hours, worms were washed off salt plates in M9 buffer containing 300 mM NaCl and transferred to normal NGM plates. After one day of recovery, the percentage of worms surviving was determined. Results for both stress assays are the average of three independent trials.

### Statistical Analysis

Survival plots were compared using the log-rank test. The maximum lifespan of a given strain was measured as the average of the lifespan of the ten longest living worms. A student's t-test was used to compare maximum lifespan between strains. Significance between strains for paraquat and juglone sensitivity assays were assessed by ANOVA. Oxygen consumption results were compared by student's t-test. Error bars show standard deviation.

## Supporting Information

Figure S1 Location of mutations in *sod* genes.
(0.02 MB PDF)

Figure S2 Heteroallelic *sod-2* mutant worms show extended lifespan.
(0.02 MB PDF)

Figure S3 *sod-2* mutant worms are sensitive to paraquat during development.
(0.02 MB PDF)

Figure S4 Mild compensatory upregulation of other *sod*­ mRNAs in *sod-sod* double deletion mutants.
(0.02 MB PDF)

Table S1 Summary of mean and maximum lifespan.
(0.01 MB PDF)
