# Supplementary material for: Deletion of the Mitochondrial Superoxide Dismutase sod-2 Extends Lifespan in Caenorhabditis elegans
Source: PLoS Genet. 2009 Feb 6;5(2):e1000361. doi: 10.1371/journal.pgen.1000361 (PMC2628729; doi:10.1371/journal.pgen.1000361)
Supplement: Figure S1 — Location of mutations in sod genes. (0.02 MB PDF) [file pgen.1000361.s001.pdf]

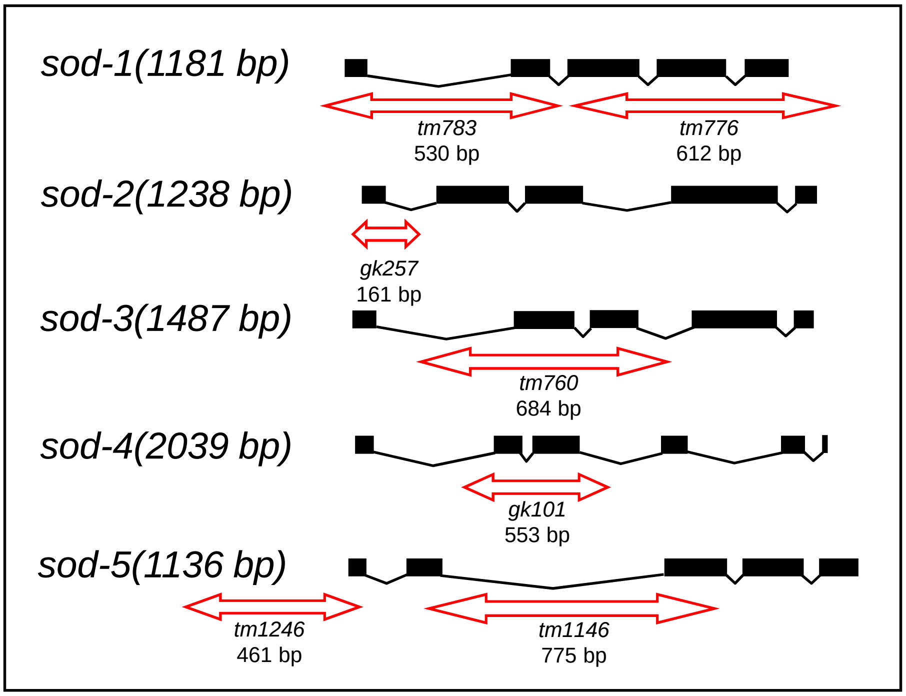

Figure S1. Location of mutations in *sod* genes. The approximate locations of the mutations in each *sod* genes is indicated by the red double headed arrow. Exons are indicated by black bars and introns with thin black lines. The size of the gene and the deletions are indicated. The *sod-2* allele *ok1030* is estimated to be 900 base pairs in size. Its location has not been identified precisely. bp = base pairs
