# Supplementary material for: Deletion of the Mitochondrial Superoxide Dismutase sod-2 Extends Lifespan in Caenorhabditis elegans
Source: PLoS Genet. 2009 Feb 6;5(2):e1000361. doi: 10.1371/journal.pgen.1000361 (PMC2628729; doi:10.1371/journal.pgen.1000361)
Supplement: Figure S2 — Heteroallelic sod-2 mutant worms show extended lifespan. (0.02 MB PDF) [file pgen.1000361.s002.pdf]

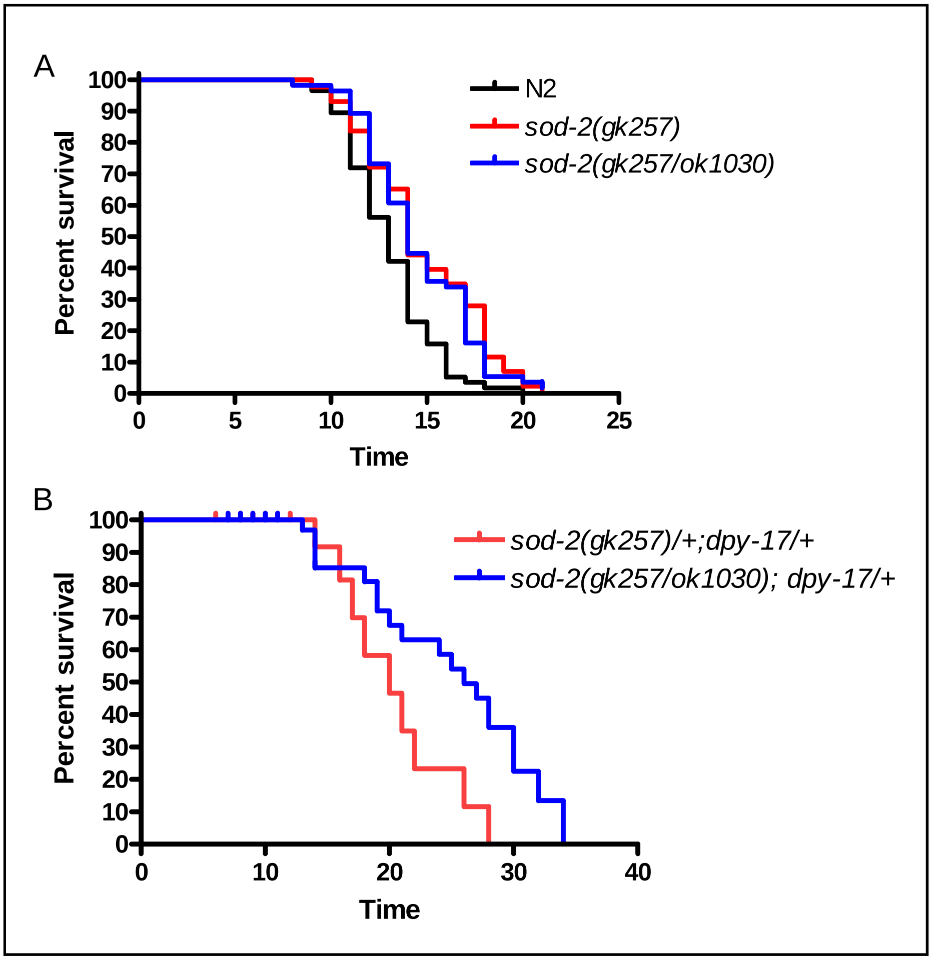

Figure S2. Heteroallelic *sod-2* mutant worms show extended lifespan. A) *sod-2(gk257)* males were crossed with *sod-2(ok1030)* hermaphrodites such that all of the resulting males would be heteroallelic *sod-2 (gk257/ok1030)* mutant worms. These worms lived as long as homoallelic *sod-2(gk257)* males and both lived longer than wild-type N2 males. B) *sod-2(gk257)* males were crossed with either *dpy-17* (control) or *sod-2(ok1030);dpy-17* hermaphrodites. Examining the lifespan of the resulting non-dumpy hermaphrodite offspring shows that *sod-2(gk257/ok1030);dpy-17* hermaphrodites live longer than *sod-2(gk257/+);dpy-17* hermaphrodites. In both cases *sod-2* mutant worms bearing both the *gk257* and *ok1030* allele together show extended lifespan suggesting that deletion of *sod-2* is responsible for the lifespan extension seen in *sod-2* homoallelic worms.
