# Supplementary material for: Deletion of the Mitochondrial Superoxide Dismutase sod-2 Extends Lifespan in Caenorhabditis elegans
Source: PLoS Genet. 2009 Feb 6;5(2):e1000361. doi: 10.1371/journal.pgen.1000361 (PMC2628729; doi:10.1371/journal.pgen.1000361)
Supplement: Figure S3 — sod-2 mutant worms are sensitive to paraquat during development. (0.02 MB PDF) [file pgen.1000361.s003.pdf]

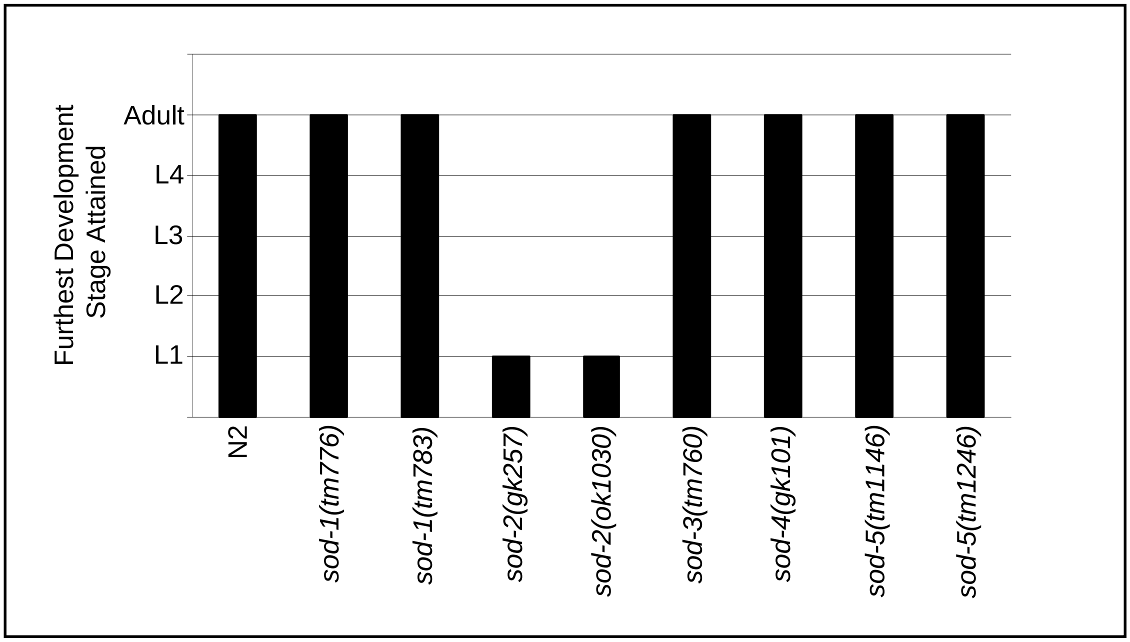

Figure S3. *sod-2* mutant worms are sensitive to paraquat during development. To assess the sensitivity of worms to oxidative stress during development, we examined the development of worms from the egg stage on 0.2 mM paraquat plates. While all of the worms showed slow development on the paraquat plates, all of the deletion mutants, except for *sod-2* mutant worms, developed to adulthood. *sod-2* mutant worms were found to arrest at the L1 stage. Thus, *sod-2* worms appear to be the most sensitive of the *sod* deletion mutants to paraquat during development.
