# Supplementary material for: Deletion of the Mitochondrial Superoxide Dismutase sod-2 Extends Lifespan in Caenorhabditis elegans
Source: PLoS Genet. 2009 Feb 6;5(2):e1000361. doi: 10.1371/journal.pgen.1000361 (PMC2628729; doi:10.1371/journal.pgen.1000361)
Supplement: Figure S4 — Mild compensatory upregulation of other sod­ mRNAs in sod-sod double deletion mutants. (0.02 MB PDF) [file pgen.1000361.s004.pdf]

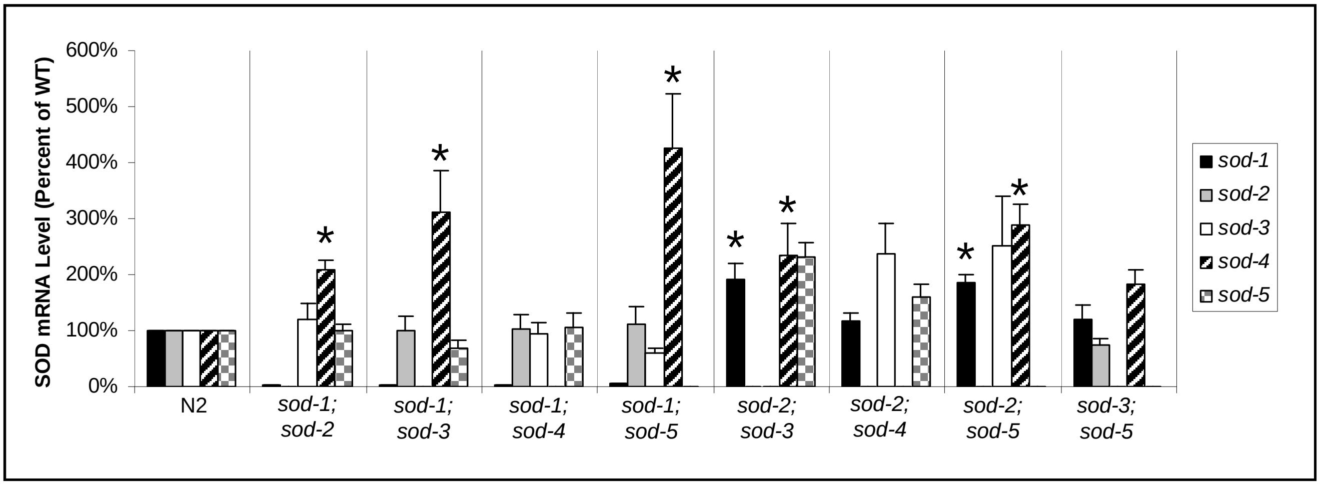

Figure S4. Mild compensatory upregulation of other *sod* mRNAs in *sod-sod* double deletion mutants. As with the *sod* single deletion mutants, there appears to be some compensatory upregulation of other *sod* genes in the *sod-sod* double deletion mutants but the magnitude of this increase is small. Specifically, all of the *sod-sod* double mutant worms (except those with a deletion in *sod-4*) show an increase in *sod-4* mRNA ranging from 2- to 4-fold. In addition, *sod-2;sod-3* and *sod-2;sod-5* mutant worms show an approximately 2-fold increase in *sod-1* mRNA. * $p<0.05$.
