# Supplementary material for: Deletion of the Mitochondrial Superoxide Dismutase sod-2 Extends Lifespan in Caenorhabditis elegans
Source: PLoS Genet. 2009 Feb 6;5(2):e1000361. doi: 10.1371/journal.pgen.1000361 (PMC2628729; doi:10.1371/journal.pgen.1000361)
Supplement: Table S1 — Summary of mean and maximum lifespan. (0.01 MB PDF) [file pgen.1000361.s005.pdf]

Table S1. Summary of mean and maximum lifespan.

Strain	Mean Lifespan	Log Rank Test vs. N2	Maximum Lifespan	P Value vs. N2	Total Death Events
N2	17.7 ± 4.0	N/A	27.4 ± 1.3	N/A	309
***sod* single deletion mutants**					
sod-1(tm776)	17.0 ± 5.5	0.97	27.9 ± 2.2	0.55	158
sod-1(tm783)	17.3 ± 3.6	0.06	26.0 ± 1.7	0.06	292
sod-2(gk257)	23.7 ± 6.6	<0.0001	38.1 ± 2.4	< 0.0001	233
sod-2(ok1030)	27.2 ± 6.5	<0.0001	43.4 ± 4.3	< 0.0001	373
sod-3(tm760)	17.1 ± 3.8	0.30	27.2 ± 2.0	0.86	109
sod-4(gk101)	19.1 ± 4.7	<0.0001	30.7 ± 2.0	0.0005	214
sod-5(tm1146)	17.2 ± 4.9	0.23	28.6 ± 3.6	0.34	176
sod-5(tm1246)	16.4 ± 4.1	0.0002	25.8 ± 1.6	0.028	216
***sod-sod* double deletion mutants**					
sod-1(tm783);sod-2(ok1030)	29.3 ± 7.7	<0.0001	46.8 ± 2.7	<0.0001	213
sod-1(tm783);sod-3(tm760)	21.0 ± 4.1	<0.0001	30.4 ± 1.9	0.0009	187
sod-1(tm783);sod-4(gk101)	17.5 ± 2.2	0.0009	22.7 ± 0.7	<0.0001	178
sod-1(tm783);sod-5(tm1246)	18.2 ± 4.5	0.28	27.5 ± 3.6	0.94	205
sod-2(ok1030);sod-3(tm760)	25.1 ± 4.6	<0.0001	34.0 ± 2.1	<0.0001	194
sod-2(ok1030);sod-4(gk101)	26.8 ± 7.0	<0.0001	41.5 ± 5.7	<0.0001	127
sod-2(ok1030);sod-5(tm1246)	24.6 ± 5.2	<0.0001	34.4 ± 0.8	<0.0001	150
sod-3(tm760);sod-5(tm1246)	20.0 ± 4.8	<0.0001	31.2 ± 2.0	0.0001	193
***sod* triple deletion mutants**					
sod-1(tm783);sod-2(ok1030); sod-4(gk101)	23.3 ± 6.2	<0.0001	43.4 ± 4.6	<0.0001	225
sod-1(tm783);sod-3(tm760); sod-5(tm1246)	18.9 ± 4.3	0.08	29.1 ± 6.6	0.44	185
sod-2(ok1030);sod-3(tm760); sod-5(tm1246)	20.3 ± 4.7	<0.0001	32.8 ± 1.8	0.0028	228
***sod-2* double deletion mutants with mutants in known lifespan extension pathways**					
daf-2(e1370)	60.8 ± 17.6	<0.0001	90.5 ± 3.5	<0.0001	168
daf-2(e1370);sod-2(ok1030)	63.5 ± 12.3	<0.0001	82.2 ± 1.9	<0.0001	159
clk-1(qm30)	29.1 ± 8.4	<0.0001	49.8 ± 2.2	<0.0001	112
clk-1(qm30);sod-2(ok1030)	43.7 ± 9.3	<0.0001	60.8 ± 4.1	<0.0001	147
isp-1(qm150)	42.3 ± 13.9	<0.0001	67.7 ± 1.9	<0.0001	114
isp-1(qm150);sod-2(ok1030)	16.9 ± 2.0	<0.0001	19.8 ± 0.9	<0.0001	69
eat-2(ad1116)	36.9 ± 8.5	<0.0001	55.9 ± 5.2	<0.0001	156
eat-2(ad1116);sod-2(ok1030)	41.0 ± 11.4	<0.0001	64.1 ± 4.4	<0.0001	111
glp-1(e2141)	32.1 ± 12.4	<0.0001	57.8 ± 3.6	<0.0001	188
glp-1(e2141);sod-2(ok1030)	38.4 ± 8.6	<0.0001	55.4 ± 2.9	<0.0001	143
